# The Physician’s Pocket Day-Book, Visiting List, Diary, and Book of Engagements

**Published:** 1859-05

**Authors:** 


ART. VIII.—*The Physician's Pocket Day-Book, Visiting List, Diary, and Book of Engagements for* 1859. Philadelphia: C. T. Price & Co.

OUR thanks are due to the publishers for this pocket-book, which will be found of convenience to the practitioner in his daily routine of visits. Among other things, it contains the code of ethics, a list of poisons and their antidotes, the Marshall Hall method for the treatment of asphyxiated persons, a list of the different remedial agents and their doses, besides other subjects of importance. It is one of the best little manuals of its kind with which we are acquainted.

